# OX40 and other immunoregulatory molecules are highly expressed on tumor infiltrating lymphocytes in oral, head and neck squamous cell carcinoma

**DOI:** 10.1186/2051-1426-2-S3-P113

**Published:** 2014-11-06

**Authors:** Ryan Montler, Richard B Bell, Rom Leidner, Marka Crittenden, Tuan Bui, Allen Cheng, Ashish Patel, Christopher Tucker, Colin Thalhofer, Andrew Weinberg

**Affiliations:** 1 Agonox, Inc, Portland, OR, USA; 2 Providence Cancer Center, Portland, OR, USA; 3 The Oregon Clinic, Portland, OR, USA

## Abstract

OX40 is a potent co-stimulatory receptor on the surface of T lymphocytes. An OX40 agonist was recently tested in a Phase I clinical trial and was found to be well tolerated and enhanced both humoral and cellular immunity in patients with metastatic cancer. Both PD-1 and CTLA-4 blockade are showing therapeutic clinical activity in late stage cancer patients. Hence, we hypothesize that the immune suppressed tumor microenvironment that characterizes oral, head and neck squamous cell carcinoma (OHNSCC) can be overcome by strategies that favor anti-tumor inflammatory T cell responses and may be an ideal tumor site for immune modulation. This investigation aims to provide a better understanding of the expression of OX40, PD-1, CTLA-4 and CD103 in OHNSCC tumor infiltrating lymphocytes (TIL). Additionally we evaluated whether phenotypic differences existed in patients with HPV positive vs. negative status.

## Methods

TIL and autologous blood were isolated from 29 patients undergoing surgery for OHNSCC. A sample of tissue was obtained from the resection specimen of the primary tumor and sent to the laboratory with autologous blood. Tumor was minced and subjected to digestive enzymes. Blood and tumor samples were separated over ficoll gradient to enrich for lymphocytes. Samples were frozen at -140°c and later thawed in batches for analysis. Proteins were then labeled with fluorescently conjugated antibodies and analyzed by flow cytometry.

## Results

The percentage of FoxP3+CD25+CD4+ (Tregs) of CD4+ T cells is significantly higher in OHNSCC Tumor Infiltrating Lymphocytes (TIL) compared to blood (p < .0005), regardless of HPV status. OX40 expression is significantly higher in the TIL Treg population relative to blood Tregs (p < .0005), also independent of HPV status. PD1 expression is increased in the Tregs (p < .0005), conventional CD4 (p=.0066), and CD8 T cells (p < .0005), in TIL relative to blood. CTLA-4 expression is increased in TIL relative to blood in all subsets with the highest expression observed in the Treg population (.0056). CD103 expression is increased on CD8 T cells in TIL relative to blood (p < .0005). CD8s from HPV positive patients have higher percentage CD103 positive cells than CD8s from HPV negative patients (p=.0056). The highest percentages of PD-1, CTLA-4 and OX40 triple positive cells were found in the Treg population of TIL compared to blood (p < .0005). Due to increased expression of OX40, CTLA-4 and PD-1 in TIL of OHNSCC patients we hypothesize that future combination clinical trials that target these pathways will be therapeutically beneficial.

